# Development and validation of the PrePain questionnaire for predicting pain

**DOI:** 10.1038/s41598-025-09044-5

**Published:** 2025-07-11

**Authors:** Filip Gedin, Andreas Hellerström, Sebastian Blome, Karin Jensen, Anna Andreasson, Maria Lalouni

**Affiliations:** 1 Department of Clinical Neuroscience, Karolinska Institutet, Nobels väg 9, D3, 17165 Solna, Stockholm, Sweden; 2 Division of Psychobiology and Epidemiology, Department of Psychology, Stockholm University, Stockholm, Sweden; 3 School of Psychology, Macquarie University, North Ryde, NSW Australia

**Keywords:** Long-term pain, PrePain questionnaire, Psychometric properties, Construct validity, Public health, Outcomes research, Epidemiology

## Abstract

The aim of this study was to evaluate the psychometric properties and construct validity of the PrePain questionnaire in both the general population and a long-term pain population. The PrePain questionnaire is an 11-item self-administered tool that assesses current pain intensity, long-term pain history, and attitudes, emotions, and behaviors related to pain using a visual analog scale (VAS). This non-randomized observational study included 200 participants: 100 individuals with long-term pain and 100 without. Participants were recruited from a specialist pain rehabilitation clinic and via social media. The study involved baseline and follow-up assessments using the PrePain questionnaire, the Short Health Anxiety Inventory (SHAI-14), and the Coping Strategies Questionnaire (CSQ). Statistical analyses included Spearman correlation for test-retest reliability, sign rank-test for sensitivity to change, Cronbach’s α for internal consistency, principal component analysis for subscale analysis, and mixed-effects linear regression for test of criterion validity. A total of 187 participants completed the study. The PrePain questionnaire demonstrated acceptable face validity and moderate test-retest reliability (coefficients ranging from 0.58 to 0.73). Internal consistency was α = 0.63. Principal component analysis suggested a two or three-factor solution. Criterion validity was supported by significant correlations between PrePain items and SHAI-14 and CSQ catastrophizing, except for the pain sensitivity item. No significant changes were observed in the clinical group between baseline and follow-up assessments. The resuts provide preliminary evidence for the reliability of The PrePain questionnaire and its validity for assessing attitudes, emotions, and behaviors related to pain in both clinical and non-clinical populations. Further research is needed to explore its sensitivity to change and utility in clinical practice.

**Supplementary Information:**

The online version contains supplementary material available at 10.1038/s41598-025-09044-5.

## Introduction

Long-term pain is a common public health problem with high prevalence in the general population^1^. It has negative consequences for society, leading to high numbers of sick leave and productivity loss^2^, and is associated with increased mortality^3^ and suicidality^4^. At an individual level, long-term pain is associated with low health-related quality of life^5^ and disability^5^. Accordingly, understanding possible mechanisms underlying the development of long-term pain, and reducing its consequences and costs, is a major public health concern.

Numerous sociodemographic factors are associated with a higher incidence of long-term pain and pain-related disability, for example, female gender^6^, old age^7^, and low socioeconomic status^8^. Additionally, long-term pain is associated with smoking^9^, obesity^10^, and psychiatric diagnoses^11^. There are several theoretical models aiming to explain the progression from acute to long-term pain, such as the diathesis-stress model^12^, the fear-avoidance model^13^, and the biopsychosocial model^14^. These models are commonly used in clinical practice, however, they offer limited empirical support for possible underlying mechanisms and etiological factors^15^. There are numerous risk factors known to contribute to the development and maintenance of long-term pain conditions, including psychosocial factors^16,17^. Also, there are several questionnaires designed to support clinical decision making and assess the risk for individuals with acute pain to develop long-term pain^18–23^. However, truly prospective data on vulnerability factors for long-term pain within a healthy population are lacking, as is a questionnaire assessing such factors.

PrePain is a prospective research project with the aim of identifying predictors for long-term pain in a pain-free population^24^. PrePain combines functional magnetic resonance imaging (fMRI) data, genetic information, and self-report questionnaires, and follow-up participants’ health outcomes via Swedish national registers. As part of the PrePain project, the self-report questionnaire was designed. Based on the literature, constructs recognized for their associations with long-term pain were represented as single items: Pain catastrophizing, health anxiety, hypervigilance, avoidance behaviors, and pain sensitivity. Pain catastrophizing is considered a maladaptive response to pain. As a concept, it generally includes a tendency of magnification, feelings of helplessness, and a lack of control when experiencing pain^25^. Among individuals with long-term pain, pain-catastrophizing and pain-related fear is associated with greater pain intensity, disability, high healthcare utilization, and poor treatment outcomes^26–28^. Health anxiety may increase the probability of developing and maintaining a long-term pain condition^29^, and is highly prevalent among individuals with long-term pain^30^. Attention or hypervigilance to pain symptoms among long-term pain patients is associated with disability, increased pain intensity, and healthcare utilization^31^. Avoidance behaviors as a coping strategy for long-term pain are associated with depressed mood and disability^32^. Increased pain sensitivity has been studied both as a characteristic of many pain conditions and as a possible vulnerability factor for long-term pain conditions^33^. The purpose of the questionnaire is to investigate if these constructs, known for their associations with long-term pain and disability, can be combined with fMRI and genetic information at baseline to predict future long-term pain. The questionnaire is easy to administer and takes less than 2 min to complete.

The aim of this study was to evaluate the psychometric properties and construct validity of the PrePain questionnaire in the general population as well as in a long-term pain population. More specifically, the aims were to evaluate feasibility, internal consistency, test–retest reliability, factor structure, and criterion validity of the questionnaire. We hypothesized a reasonable test–retest reliability and an internal consistency and factor structure that would support the use of the individual items separately. In addition, we hypothesized that all items would correlate with health anxiety and pain catastrophizing based on the established association between health anxiety, pain catastrophizing, and long-term pain.

## Method

This study employed a non-randomized observational design, aiming to include approximately 200 participants: a clinical group with 100 individuals experiencing long-term pain and a non-clinical group with 100 individuals without long-term pain. The study protocol was preregistered in OSF (https://doi.org/10.17605/OSF.IO/WPH47).

### Study participants and recruitment

Participants in the clinical group were adults recruited from a specialist pain rehabilitation and orthopedic surgery clinic in Region Stockholm, Sweden. The patients at this clinic were medically assessed to ensure they met the criteria for long-term pain (pain > 3–6 months). No additional medical examination was deemed necessary for inclusion in the clinical group. The patients were given information about the study via their rehabilitation groups and via posters in the reception area. Participants in the non-clinical group were recruited via advertisements on social media platforms (Facebook and Instagram). The advertisement emphasized that participants should be free from long-term pain. Participants in both groups received a weblink to the study. All participants received two vouchers for cinema tickets after completing their last assessment.

### Ethical approval and consent to participate

All methods were carried out in accordance with relevant guidelines and regulations. The study protocol was approved by the Swedish Ethical Review Authority (Dnr: 2023-03275-02). Informed consent was obtained from all participants prior to their inclusion in the study. All participants provided written informed consent through a secure digital platform.

### Procedure

The informed consent, assessments and questionnaires were self-administered digitally via a secure online platform (REDCap), comprising an initial survey and a follow-up survey 14 days later. The baseline assessment included general demographic questions (age, sex, education), the PrePain questionnaire, the Short Health Anxiety Inventory (SHAI-14), and the Coping Strategies Questionnaire (CSQ). Fourteen days after completing the initial survey, participants received an email with a weblink to the follow-up survey, which included the same set of assessments (Pre-Pain questionnaire, SHAI-14, and CSQ). Up to three daily reminders were sent to encourage participation in the follow-up survey.

### Questionnaires

#### The PrePain questionnaire

The PrePain questionnaire is an 11-item self-administered questionnaire (appendix 1). It consists of 3 parts, starting with a question about whether the participant has current pain. If yes, the participant is asked to grade their pain intensity at different body sites on a visual analog scale (VAS), with endpoints defined from “no pain” to “worst imaginable pain”. The second part of the questionnaire consists of 4 questions y/n about long-term pain: current long-term pain, previous episodes of long-term pain, family history of long-term pain, and worry about developing long-term pain. Finally, the questionnaire contains 6 dimensional questions about attitudes, emotions and behaviors in relation to pain and illness: perceived pain-sensitivity, avoidance of possibly painful situations, distressed when in pain, relief when pain subsides, focus of bodily symptoms, and negative thoughts and anxiety. The questions are graded using a VAS, 0–100 mm, with endpoints (and for some items mid points) defined (see appendix 1).

#### Short health anxiety questionnaire (SHAI-14)

The Short Health Anxiety Inventory (SHAI-14) is a self-report questionnaire, assessing health anxiety^34^. SHAI-14 is a shorter version of the Health Anxiety Inventory (HAI). consisting of 14 items scored from 0 to 3, and the total score is calculated by summing the item scores, resulting in a range from 0 to 42. SHAI-14 has demonstrated a reliable internal consistency (α = 0.81–0.89), a good test–retest reliability (r = 0.87)^34–36^, and divergent validity, as physically ill individuals did not score higher on SHAI-14 than healthy individuals, and further, to distinguish individuals suffering from health anxiety from those with other anxiety disorders^34^.

#### Coping strategies questionnaire (CSQ)

The Coping Strategies Questionnaire (CSQ) is a self-report questionnaire originally developed to measure coping strategies in individuals with long-term pain^37^. The questionnaire consists of 48 items scored on a 6-point Likert scale, and the total score is calculated for each subscale resulting in a range from 0–36. In a Swedish sample, the internal consistency of the CSQ was good, but the test–retest reliability showed greater variability^38^. For the purpose of this study, the subscale Catastrophizing was used as a criteria variable.

### Analysis and statistics

The questionnaire’s face validity was examined via a convenience sample, recruiting 10 individuals who consented to complete the questionnaire. After completion they were interviewed regarding the questionnaire, asking if the included items were easy to comprehend and without ambiguities, ensuring the items were suitable.

Test–retest reliability in the non-clinical sample was first assessed using Spearman correlations between baseline and the two-week follow-up for each item separately. A non-parametric test was chosen due to the non-normal distribution of the variables, and a Spearman coefficient of 0.7 was used as a threshold for acceptable reliability. In addition, Intraclass Correlation Coefficients (ICCs) were calculated using a two-way random-effects model with absolute agreement to provide a more robust estimate of reliability. ICCs were computed for both the non-clinical and clinical samples. The inclusion of ICCs complements the Spearman analysis and allows for comparison with psychometric standards.

Sensitivity to change in the clinical sample was tested using sign rank-test (non-parametric pairwise test) for each item separately comparing baseline and follow-up assessments on an individual level. *P* < 0.05 was used as a cut-off for a significant reduction.

Cronbach’s α was calculated as a measure of internal consistency on the baseline assessments on all participants. Because the different items are thought to measure different constructs, the Cronbach’s α was expected to be relatively low.

Principal component analysis (varimax rotation and eigenvalue of 1) was performed to evaluate the component structure of the questionnaire. The items are thought to measure different constructs relating to pain, why we did not expect to find a component structure that supports the use of a summary score or subscales.

Criteria validity was tested by (1) analyzing the difference in each PrePain questionnaire item between individuals with pain and individuals without pain and (2) correlating all VAS scale ratings with SHAI-14 score and CSQ catastrophizing subscale. (1) Mixed-effects linear regression bootstrapped with 2000 repetition adjusted for age and sex was used to calculate the differences between participants in the clinical and the non-clinical groups for each item. (2) Mixed-effects linear regression bootstrapped with 2000 repetition adjusted for age and sex was used to calculate the association with each item and all criteria variables. A *p*-value of 0.05 was used as a cut off for statistical significance.

Criteria validity analysis was conducted in STATA 18. All other statistical analyses were performed in SPSS 28.

## Results

In total, 187 individuals were included in the data-analysis: 76 individuals from the clinical group (age-range 19–71 years) and 111 individuals from the non-clinical group (age-range 21–77 years), all of whom completed the questionnaire at baseline and at the two-week follow-up. A total of 153 individuals were excluded from the analysis because they did not complete the initial or follow-up assessment despite several reminders.

Details of demographics in all samples are presented in Table 1. A majority of the participants had current pain, 91% in the clinical group and 55% in the non-clinical group. Further, 93% in the clinical group and 37% in the non-clinical group reported suffering from long-term pain. Since it was not possible to verify whether the people in the non-clinical group who reported long term pain had a formal diagnosis or met the criteria for long-term pain, they were included in the data-analysis.

**Table 1:** Participant demographics.

	Clinical n = 76 n (%)	Non-clinical n = 111 N (%)
Male	26 (34.2)	43 (38.7)
Female	50 (65.8)	65 (58.6)
Other	0	3 (2.7)
Total	76	111
Age (mean)	45.68	41.49
Age (range)	21–71	21–72
No high school degree	2 (2.6)	1 (0.9)
High school, 2 years	6 (7.9)	4 (3.6)
High school, 3 years	17 (22.4)	11 (9.9)
Higher education < 3 years	16 (21.1)	15 (13.5)
Higher education > 3 years	35 (46.1)	80 (72.1)
Pain now	69 (91)	61 (55)
Long-term pain	71 (93)	41 (37)
Previous pain	67 (88)	52 (47)
Family pain	40 (56)	40 (36)
Fear of pain	64 (84)	40 (36)

### Face validity

The individuals in the face validity test who completed the questionnaire confirmed that the questionnaire was clear and easy to comprehend, thus indicating an adequate face validity.

### Test–retest

The average duration between test and retest was 15.26 days (SD = 5.03) in the non-clinical group. Test–retest reliability was first assessed using Spearman correlations for each item, with coefficients ranging from 0.58 to 0.73. Specifically, the coefficients were 0.67 for pain sensitivity, 0.62 for pain avoidance, 0.73 for pain unpleasantness, 0.60 for pain relief, 0.58 for body focus, and 0.60 for pain anxiety. In addition, ICC values for individual ratings ranged from 0.58 to 0.72, indicating moderate to good reliability^39^. In the clinical sample, ICC values ranged from 0.36 to 0.61, suggesting poor to moderate reliability. These results support the temporal stability of the PrePain items, particularly in the non-clinical group. Full ICC values and confidence intervals are presented in Tables 2 and 3.

**Table 2:** Summary of ICC results (non-clinical sample).

	ICC (Individual)	95% CI	Interpretation (Koo and Li)
Sensitivity	0.675	0.560–0.765	Moderate to good
Avoidance	0.580	0.439–0.692	Moderate
Unpleasantness	0.715	0.611–0.795	Good
Pain Relief	0.618	0.488–0.721	Moderate
Body focus	0.579	0.442–0.690	Moderate
Anxiety	0.594	0.459–0.702	Moderate

**Table 3:** Summary of ICC results (clinical sample).

	ICC (Individual)	95% CI	Interpretation (Koo and Li)
Pain sensitivity	0.586	0.418–0.716	Moderate
Avoidance	0.410	0.204–0.582	Poor to moderate
Pain unpleasantness	0.365	0.157–0.543	Poor to moderate
Pain relief	0.611	0.448–0.734	Moderate
Body focus	0.400	0.193–0.573	Poor to moderate

### Sensitivity to change

No significant change was found in the clinical group in any of the six items between baseline and follow-up assessments two weeks after.

### Internal consistency

The internal consistency for the six-dimensional questions was α = 0.63. Component-to-total score correlations ranged between α = 0.53–0.67.

### Subscale analysis

The principal component analysis reported different solutions in the clinical and in the non-clinical group and in the total sample. In the clinical group, three components attained an eigenvalue > 1.0, with pain unpleasantness, body focus and pain anxiety included in the first component, pain sensitivity and avoidance in the second component and item pain relief in the last component. In the non-clinical sample, two components attained an eigenvalue > 1.0, with item avoidance, body focus and pain anxiety as the first component, and item pain sensitivity and pain relief as a factor with item pain unpleasantness showing a strong cross loading across the two components. In the combined study sample, a two-factor solution was again suggested, with pain sensitivity alone constituted on component and the other items the second component.

### Criteria validity

As shown in Table 4, the PrePain questions were generally associated with both health anxiety and pain catastrophing, supporting the construct criterion validity. Significant correlations were observed for questions related to the avoidance of painful situations, unpleasantness when in pain, vigilance to bodily sensations, and negative thoughts during painful episodes. However, the question about feeling relieved when the pain subsides did not correlate with health anxiety. The question regarding pain sensitivity did not correlate with any of the criterion variables.

**Table 4:** PrePain criterion validity correlations with SHAI and CSQ, age and sex.

PrePain item	CSQ		SHAI-14		Age		Sex	
	b (95% CI)	*p*-value	b (95% CI)	*p*-value	b (95% CI)	*p*-value	b (95% CI)	*p*-value
Sensitivity	0.010 (0.026–0.045)	0.595	0.015 (− 0.041–0.012)	0.283	0.167 (0.031–0.302)	0.016	2.86 (0.707–6.444)	0.116
Avoidance	− 0.057 (− 0.093– − 0.008)	0.002	− 0.043 (-0.065– − 0.021)	0.000	0.090 (− 0.056 − 0.237	0.227	1.739 (− 2.518–5.996)	0.423
Unpleasantness	− 0.135 (− 0.189– − 0.081)	0.000	− 0.050 (− 0.081–0.019)	0.002	0.176 (0.032– 0.320)	0.017	1.35 (− 2.349–5.045)	0.475
Pain relief	− 0.434 (− 0.083– − 0.004)	0.033	− 0.004 (− 0.030–0.023)	0.791	− 0.205 (− 0.346– − .065)	0.004	3.604 (0.097–7.111)	0.044
Body focus	− 0.051 (− 0.086–0.014)	0.007	− 0.069 (− 0.097 − − 0.040)	0.000	0.162 (0.033–0.290)	0.014	2.314 (− 1.213– 5.841)	0.198
Anxiety	− 0.110 (− 0.159– − 0.061)	< 0.001	− 0.091 (− 0.120 − − 0.062	0.000	0.205 (0.071–0.339)	0.003	3.825(0.310–7.339)	0.033

## Discussion

The PrePain questionnaire is a 11-item questionnaire developed for a prospective study with the aim to find risk-factors for long-term pain (Under review Gedin et al., 2024). The PrePain questionnaire represents a short version of several constructs that have previously been associated with long-term pain, and aims to explore healthy individuals’ attitudes, emotions and behaviors in relation to pain. Here, we assessed the psychometric properties of the PrePain questionnaire in a clinical and a non-clinical sample.

There were differences between the clinical and non-clinical groups in every item of the questionnaire, supporting its criterion validity. Individuals in the clinical sample reported an increased likelihood of avoiding painful stimuli, feeling unpleasantness, and having negative thoughts when in pain. They also reported being more vigilant about bodily sensations compared to the non-clinical group. These findings were expected based on previous research, which shows that health anxiety is common among individuals with long-term pain^30^. Interestingly, individuals in the clinical sample perceived themselves as less sensitive to pain than the non-clinical group. This was unexpected since numerous studies have demonstrated that individuals with long-term pain are more pain-sensitive than pain-free individuals when exposed to experimental pain^40^, and that perceived pain sensitivity is associated with a lower pain threshold^41^. On the other hand, it has been shown that subjective pain sensitivity correlates poorly with objective measures of pain threshold^42^. Pain sensitivity can be affected by situational factors, such as sleep deprivation or negative cognitions^43,44^. However, it is neither likely nor possible to postulate any such biases between the groups. The prevalence of family members with long-term pain was higher in the clinical group, which is supported by research on the genetic influence of long-term pain^45,46^.

The results confirm that, with a few exceptions, the PrePain questionnaire is correlated to the expected criteria variables, both in the clinical and the non-clinical sample. The question about pain sensitivity was an exception. No correlations were found between this question and the criteria variables in any of the groups. The question about feeling relieved when the pain goes away was correlated with the subscale Catastrophizing in CSQ, but not with SHAI-14. A possible explanation to this finding could be that individuals with higher levels of health anxiety are hyper-aware of unusual or new bodily sensations, which may prevent them from feeling relief when the pain subsides.

The PrePain test–retest reliability coefficients were in the range of 0.58–0.73, which is considered questionable/acceptable. Only one question/item showed a coefficient exceeding 0.7. In the face validity analysis, the questions were regarded as clear and easy to comprehend. One reason for the low test–retest reliability could be that the questions do not capture stable personality traits, but rather states influenced by situational factors. Another reason for the moderate test–retest reliability might be due to technical issues. The questions were answered digitally by moving a marker on a VAS-scale. It is not known, but probable that many of the participants used a smartphone device to answer the questions. Since most smartphone screens are relatively small, it’s hard to make fine-tuned answers.

The internal consistency was, as expected, not better than acceptable (α = 0.63). The results confirm the purpose of the PrePain questionnaire, to address distinctive constructs which could all add to the vulnerability for long-term pain. In particular, the questions about pain sensitivity and pain relief demonstrate a low co-variation with the other questions, indicating that these questions represent different constructs. The principal component analyses did not yield a consistent component structure. All suggested solutions included at least two components, and the structure varied between the samples. Therefore, a single-component solution, i.e., using a total scale summary score for the PrePain questionnaire, is not supported. Likewise, there is no evidence to support a subscale structure. Taken together, the PrePain questionnaire should be used as six individual items.

A majority of the participants in the clinical group were in different phases of a rehabilitation program with an average duration of 14 weeks. We were interested if any treatment effects would be visible in the test–retest assessments for this group. There were, however, no significant reductions in any of the items. Since we had no knowledge of the temporal occurrence of possible treatment effects in the rehabilitation program, and since the baseline and follow-up measures only had a duration of two weeks, this result is not surprising.

The psychological constructs included in the PrePain questionnaire were selected based on previous research showing their relevance to long-term pain, and because they could be measured with short self-report questions. We focused on constructs like pain catastrophizing, health anxiety, and avoidance behavior, which are supported by the literature and suitable for use in both clinical and general populations. We recognize that other factors—such as expectations, threat value of pain, and cognitive flexibility—are also important, but were not included in order to keep the questionnaire short and easy to use in large-scale studies. While avoidance behavior can be complex, we aimed to capture a general tendency rather than a detailed behavioral pattern.

A limitation of this study is the characteristics of the participants, which show a bias towards a high education level and female gender. Specifically, 78 percent of the participants have a college degree, and 63 percent are female. However, the general population also has a high proportion of individuals with higher education, about 45%, and there are more women with long-term pain than men^2,47^. Another limitation is the rather small sample size for the PCA. As previously mentioned, a fairly large number of the participants in the non-clinical group (37%) stated that they had long-term pain. They were included in the analysis under the assumption that we didn’t know if these individuals actually fulfilled the criteria for a pain diagnosis. This might have affected the outcomes in the non-clinical group. However, long-term pain is very prevalent in the general population, with approximately 20% experiencing long-term pain at any given moment^1,48^. The non-clinical group might thus be close to a representative sample.

The item labeled “pain anxiety” in the PrePain questionnaire was intended to reflect cognitive and emotional responses to pain, including worry about health deterioration. While this overlaps with aspects of pain catastrophizing, we acknowledge that the construct is broader and typically includes dimensions such as rumination, magnification, and helplessness. Capturing this complexity with a single item is a limitation. Our aim was to include brief indicators of several relevant constructs, but future versions of the questionnaire may benefit from a more precise representation of pain catastrophizing, in line with ongoing discussions in the field.

Several potential questions for future research arise from this study. For example, the sensitivity to change was not systematically examined. Thus, it could be relevant to explore the questionnaire’s sensitivity to change during a pain treatment. It would also be valuable to examine its usefulness in clinical practice and in clinical studies. It might be worthy to investigate if the modest test–retest reliability would improve if the tests were completed on paper or on a standardized computer screen. It might also be useful to further examine the qualities of the question of pain sensitivity, both psychometrically by using another criteria variable, for example the Pain Sensitivity index^49^ but also to investigate its correlation with experimentally induced pain, in clinical and non-clinical groups.

In conclusion, this is the first study investigating the psychometric properties of the PrePain questionnaire. The data from the present study provides preliminary evidence in support of the reliability and validity of the PrePain questionnaire. The Pre-Pain questionnaire seems to have utility as a self-report questionnaire to assess healthy individuals’ beliefs, behaviors, and attitudes to pain in a couple of minutes.

## Electronic supplementary material

Below is the link to the electronic supplementary material.

Supplementary Material 1.

## Data Availability

The data that support the findings of this study are available from the corresponding author, Filip Gedin, upon reasonable request.
