# Supplementary material for: Development and validation of the PrePain questionnaire for predicting pain
Source: Sci Rep. 2025 Jul 11;15:25112. doi: 10.1038/s41598-025-09044-5 (PMC12254292; doi:10.1038/s41598-025-09044-5)
Supplement: Supplementary file 1 — Supplementary Material 1. [file 41598_2025_9044_MOESM1_ESM.docx]

**Supplementary material -** Appendix 1

# Current Pain

• Do you have pain anywhere in your body right now? 🞎YES 🞎NO If yes, where? Please place a clear cross and select the intensity:

**No pain Worst imaginable pain**

Head [ ]

Jaw [ ]

Neck [ ]

Back [ ]

Shoulder [ ]

Throat [ ]

Abdomen [ ]

Arms [ ]

Legs [ ]

Hip [ ]

Feet [ ]

# Long-term Pain

*Long-term pain is the type of pain that cannot be explained by temporary headaches, muscle soreness, menstrual cramps, or similar conditions. Long-term pain lasts for more than three months continuously.*

- Are you currently experiencing long-term pain? 🞎YES 🞎NO
- Have you experienced periods of intense pain in your body? 🞎YES 🞎NO
- Do any close relatives suffer from long-term pain? 🞎YES 🞎NO
- Are you concerned about developing long-term pain? 🞎YES 🞎NO

# What it is usually like

- How pain sensitive do you perceive yourself to be compared to others, 0-100 mm: Place a clear cross on the line.

[ ]

Very pain sensitive normal very pain tolerant

***Please turn the page!***

- To what extent do you avoid situations due to the risk of experiencing pain (e.g., tattoos, playing football, dental visits):

[ ]

Always sometimes never

- How distressing do you find it when you are in pain?

[ ]

Very distressing Not at all distressing

- How relieved do you feel when the pain subsides?

[ ]

Very relieved Not at all

- In general, how much do you focus on your body and its reactions when you believe you are sick?

[ ]

Very much Not at all

- Does pain usually lead to negative thoughts about your health worsening?

[ ]

Always sometimes never
